# Correction: Pazzini et al. Mechanical Characterization of Thin Asphalt Overlay Mixtures with 100% Recycled Aggregates. *Materials* 2023, *16*, 188

**DOI:** 10.3390/ma18225137

**Published:** 2025-11-12

**Authors:** Margherita Pazzini, Giulia Tarsi, Piergiorgio Tataranni, Claudio Lantieri, Giulio Dondi

**Affiliations:** Department of Civil, Chemical, Environmental and Materials Engineering (DICAM), University of Bologna, 40131 Bologna (BO), Italy

Following publication, concerns were raised regarding the peer-review process related to the publication of this article [1]. Adhering to our standard procedure, the Editorial Board conducted an investigation, which determined that, while the peer-review process does comply with MDPI’s Editorial process policy (https://www.mdpi.com/editorial_process), the contribution of one of the three reviewers does not comply with MDPI’s Guideline for Reviewers (https://www.mdpi.com/reviewers#_bookmark11) or the expectations of the Editorial Board. As a result, the Editorial Board has decided to remove the contribution of Reviewer 3 from the open peer-review record (https://www.mdpi.com/1996-1944/16/1/188/review_report). Following discussion with the authors, the recommended citation was removed from this publication [1].

The reference [5] in the original publication, Raza, A.; Khan, I.; Tufail, R.F.; Frankovska, J.; Mushtaq, M.U.; Salmi, A.; Awad, Y.A.; Javed, M.F. Evaluation of Moisture Damage Potential in Hot Mix Asphalt Using Polymeric Aggregate Treatment. *Materials* **2022**, *15*, 5437, has been removed. With this correction, the order of some references has been adjusted accordingly.

The authors state that the scientific conclusions are unaffected. This correction was approved by the Academic Editor. The original publication has also been updated.
